# Hemostatic Net Versus Surgical Drain After Deep Plane Facelift Surgery: A Prospective Randomized Controlled Trial

**DOI:** 10.1007/s00266-025-04745-8

**Published:** 2025-03-25

**Authors:** Maram Ismail, Samir Ghoraba

**Affiliations:** 1 Plastic and Reconstructive Surgery Department, Cairo University, Cairo, Egypt; 2 Plastic and Reconstructive Surgery Department, Faculty of Medicine, Tanta University, Tanta, Egypt; 3 The Corner Mall, Al Sadat Axis, New Cairo 1, Cairo, Egypt

**Keywords:** Deep plane facelift, Facelift surgery, Aesthetic surgery, Facial rejuvenation, Hemostatic net, Hematoma

## Abstract

**Background:**

Several studies have discussed the safety and effectiveness of the hemostatic net after facelift surgery. However, fearful of having to deal with a hematoma or seroma following the surgery, many surgeons opt to leave surgical drains in place for a short time after the procedure. There is minimal data from studies comparing the hemostatic net to surgical drains.

**Objectives:**

To compare the efficacy of surgical drains and the hemostatic net after deep plane facelift surgery.

**Materials and Methods:**

This study prospectively compares the effectiveness of both methods in a randomized controlled approach, including a consecutive series of 160 patients who underwent deep plane facelifts throughout a 6-month period. We compared the incidence of hematoma, seroma, edema, and other complications during the postoperative period using surgical drains and the hemostatic net.

**Results:**

Eighty female patients were included in each group. Analysis of the postoperative data showed no significant difference in hematoma and seroma rates between the drain and net groups. Both methods were associated with comparable degrees of postoperative edema (*p* =  0.737). The occurrence of other complications such as ecchymosis, congestion, and necrosis did not show a significant correlation to either method.

**Conclusions:**

The hemostatic net and surgical drain have comparable outcomes in terms of controlling hematoma and seroma formation after deep plane facelift surgery and show similar degrees of postoperative swelling.

**Level of Evidence I:**

A randomized controlled trial.

This journal requires that authors assign a level of evidence to each article. For a full description of these Evidence-Based Medicine ratings, please refer to the Table of Contents or the online Instructions to Authors www.springer.com/00266.

## Introduction

Hematoma and seroma are considered the most serious complications following facelift surgery, they can lead to unfavorable outcomes such as infection, frank tissue necrosis, and delayed healing of the skin flap [6, 9, 10].

The reported incidence of hematoma rates ranges from 0.6 to 14.2%. [1–3]. Patient-related factors, including male sex, pre-existing hypertension, nonsteroidal anti-inflammatory intake, and smoking, have been frequently identified in the literature as variables that raise the chance of hematoma formation. Procedure-specific factors include intraoperative blood loss and increased perioperative peak systolic blood pressure [1, 4, 5].

Numerous researches have already addressed the effectiveness of compression dressings, tissue sealants, and perioperative blood pressure management in lowering the risk of these complications [4, 6–8].

Auersvald and Auersvald initially utilized the hemostatic net by quilting the skin flap raised during their facelift procedure to reduce the dead space in the subcutaneous plane, significantly decreasing their hematoma rate from 14.2 to 0% [9].

Comparable outcomes have been found with the hemostatic net application after facelift surgery in further interventional studies, including a 5-year experience by Janssen et al. [11, 12]. Furthermore, the safety of the net is now proven by literature, especially when investigating its effect on flap viability. The published experience of surgeons using the net suggests that it lowers the rates of hematomas without raising the risk of developing ischemia or necrosis [9, 13, 14].

The net technique also provides an additional technical benefit as the skin flap can be positioned more precisely, improving skin redraping and enhancing the definition of the jaw lines [15].

Postoperative suction drains have been instinctively used with facelift surgery following the anticipation that they reduce the risk of these complications. Although evidence from the literature does not strongly support this assertion [6, 16–18], many surgeons still continue to favor their use. This preference may be explained by the advantage of continuous drainage and monitoring of the accumulated fluid that suction drains provide. Additionally, it is worth noting that applying the hemostatic net is relatively time-consuming as it can take up to 30 min to be applied [11].

Many research studies have proved the effectiveness of the net utilization following facelift surgery [9, 11, 12, 15]. However, no evidence has precisely compared it to the widely used suction drain. This study compares the two methods in a randomized controlled setting with an in-depth evaluation of a wide set of variables. Additionally, it carefully examines the degree of postoperative edema primarily linked to each technique, which can significantly impact the surgical outcome and patient satisfaction [19–21].

## Materials and Methods

A consecutive series of 160 female patients who underwent deep plane facelift surgery were randomized to two groups throughout 6 months between January 2024 and June 2024. For each method, a group of 80 participants were included. Randomization was performed using a computer-generated sequence by a blinded team member. Simple randomization technique was employed. Participants were assigned and allocated to treatment groups based on the generated sequence numbers sealed in opaque envelopes. Hypertension, the use of blood thinners, and smoking were recorded as risk factors, and a potential relation to the rate of complications was investigated using the two-tailed Spearman correlation test. During the postoperative period, the incidence of hematoma, seroma formation, and other complications was inspected and a comparison between both groups was made. A detailed analysis of the degree of postoperative edema was done using a classification of three grades with the help of the grading used by Marchac and Greensmith [22]. We described grade 1 for minor edema, grade 2 for a moderate amount, and grade 3 for marked or unusual edema.

Participants were also assessed for the presence of other complications such as ecchymosis, congestion, necrosis, or skin irregularities. Informed consent was obtained from all patients. Group 1 comprised 80 patients for which surgical drains were inserted at the end of the surgery and a follow-up of the amount and character of fluid collected was done. The drains were removed after 48 h in average. The hemostatic net was applied to 80 other patients in group 2 and removed after 48 h during the first follow-up visit.

Patients were hospitalized for the first 24 h postoperatively. Patients were checked on days 1, 2, and 6 and for at least 2 weeks after the procedure. At every assessment, the presence of hematoma, seroma, and postoperative edema was observed. Any additional complications were also recorded.

### Anesthesia Technique

General anesthesia was used for all patients included in the study. Induction was done by intravenous propofol, a total of (100–200 mg), fentanyl (100 mcg), atracurium (0.3–0.4 mg/kg), and midazolam (5 mg). This was followed by isoflurane (1–5%), propofol (0.1–0.2 mg/kg/min), and dexmedetomidine hydrochloride (0.7 mcg/kg/hr) for maintenance.

### Surgical Technique

All procedures were performed by a single surgeon. Prior to incision, tumescent was infiltrated subcutaneously with the use of 20 mL of lidocaine 2%, 200 mL of saline solution 0.9%, 200 mg of tranexamic acid, and epinephrine 1:200,000.

Through classical facelift incision, minimal skin undermining was performed to allow entry to the deep plane. The entry point was always between the junction of fixed and mobile superficial musculoaponeurotic system (SMAS), which was about 2 cm in front of the ear lobule along a line extending from the lateral canthus to the angle of the mandible. A sharp dissection was used to create the entry to the deep plane. After that, blunt dissection was performed through the facial spaces to protect the facial nerve branches. The masseteric space was always the first to be dissected, followed by the buccal space, and lastly, the prezygomatic space. This blunt dissection would expose the facial ligaments. The masseteric, the zygomatic, and the cervical retaining ligaments were sharply released with caution to protect the adjacent facial nerve branches.

The lateral platysmal edge was sharply dissected off the anterior border of the sternomastoid with great caution to protect the external jugular vein and the great auricular nerve. The SMAS-platysmal flap is bluntly dissected until the facial pulsation is detected. The tail of the parotid is mobilized off the mastoid process to deepen the gonial angle.

The composite flap is anchored to the mastoid fascia behind the auricle, and to the fixed SMAS in front of the auricle using 0 Vicryl suture after vertical vectoring. The patients were instructed to avoid sleeping on the side of the face for 2 weeks.

For group 1, placement of suction drains was done through the end of the posterior auricular incision in a subcutaneous plane, advanced to just before the mid-cervical line in patients where a complete face and neck lift was done or to the end of the dissection line in cases where less advancement with the dissection was needed. A closed suction unit of 12 Fr was used and usually removed after an average of 2 days.

**Fig. 1:**
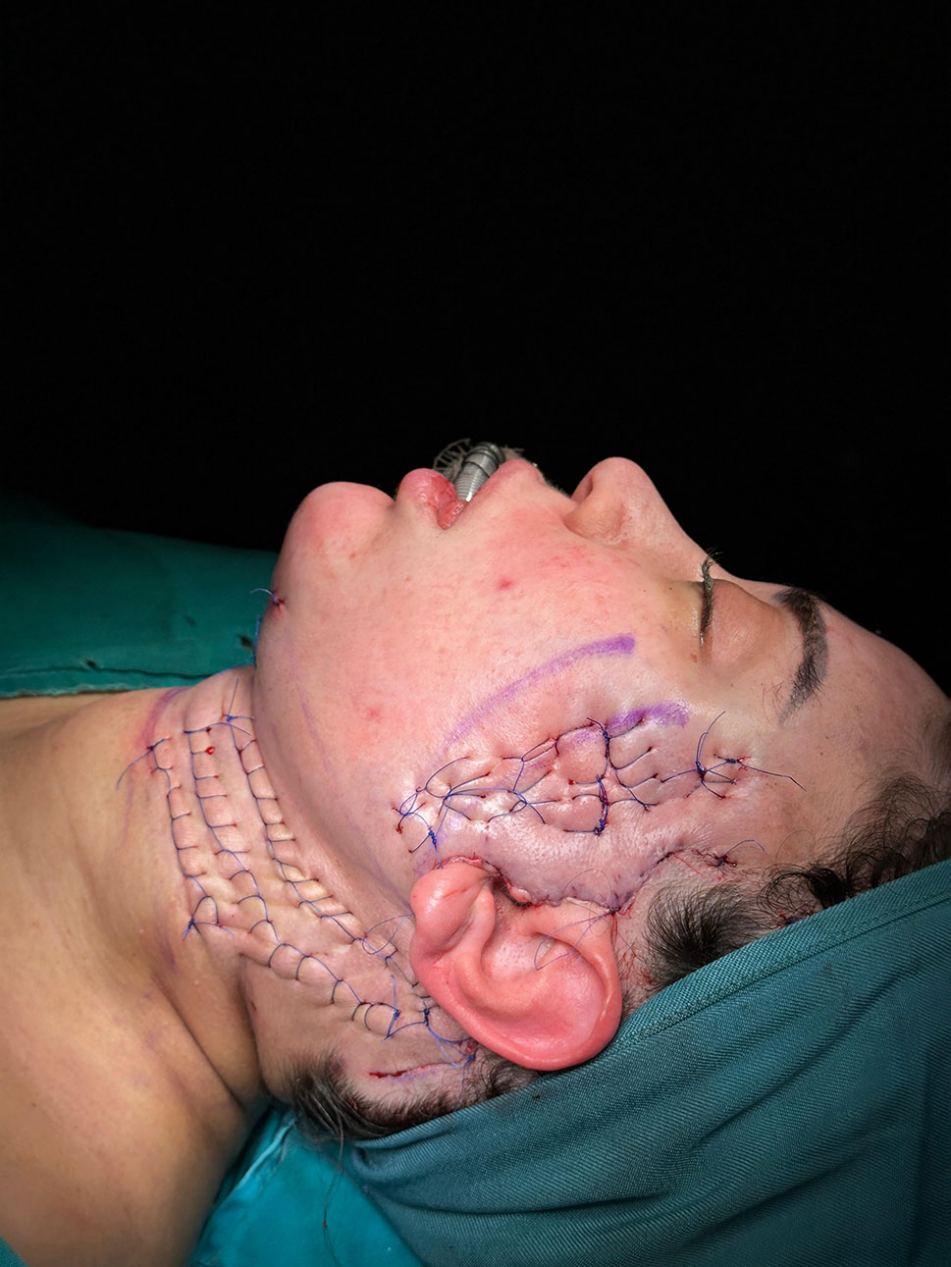
Intraoperative application of the hemostatic net

For group 2, a 3/0 Prolene suture was used to create the hemostatic net. The initial line was placed beneath the mandibular border, and additional lines were added in a parallel pattern as needed. After the closure of the skin flap while the head is in the left-lateral position, the hemostatic net is applied on the right side and vice versa. The needle route follows a consistent pattern, entering the skin perpendicularly, transfixing at a 45-degree angle into the platysma, and emerging at the same angle approximately 1.0 cm from the point of entrance (Fig. 1). More parallel lines were performed according to the width of the dissected area. A gentle traction is maintained by the assistant to ensure that the sutures’ tension would not be too loose or excessively tense, hindering the blood circulation. This closes the dead space between the skin and the platysma minimizing the room for any fluid collection. After 48 h, the net sutures were taken out.

### Statistical Methodology

The sample size was calculated using power and sample size calculator for clinical study, with 0.05 alpha error, CI of 95%, and a non-inferiority margin of 2. The study was powered at 80% to detect clinically significant differences.

SPSS version 21 was used for data analysis. Both parametric and nonparametric tests for significance were performed, using chi-square with Fisher exact for qualitative data and Student’s t test for parametric normally distributed data. Spearman correlation analysis was done to correlate the results to the risk factors and type of surgery.

P equal to or less than 0.05 was the threshold for significance.

## Results

A total of 160 facelifts were performed with each group comprising 80 female patients. The majority of patients were middle-aged, with a mean age of 45.5 years. The mean age of the drain and net groups was 46.3 and 44.7, respectively. Classical facelift was done for 56.8%, while 32% had an additional open neck lift through a submental incision. A total of 10.6% of the study participants required the midface only to be lifted. No significant correlation was found between different age groups and the type of surgery performed.

Assessment of the potential risks for developing complications revealed a 9.4% rate of hypertension among the study participants, and 8.1% were smokers. No prevalence for risk factors related to either group of the study was detected. Spearman’s two-tailed correlation coefficient test was used to study the relationship between the different risk factors including smoking and the development of postoperative complications.

### Primary Outcomes

#### Hematoma

Throughout the entire study, there was an occurrence of one hematoma among the net group, it measured approximately 7 × 5 cm in the left side of the face and it was located in the sub-SMAS layer. It was observed 12 h after the surgery and surgical evacuation with bleeding control was done with no reported recurrence. This incidence of hematoma formation did not represent a significant correlation.

#### Seroma

Seven patients in each group developed postoperative seroma (8.8%) which means there was no difference in seroma rate between both groups. The mean volume of the aspirated fluid measured 14 ± 10 ml with the peak volume detected within the first 7 days postoperatively. 71% of them were located in the submental region, while the remaining 28.5% were postauricular.

#### Postoperative Edema

**Fig. 2:**
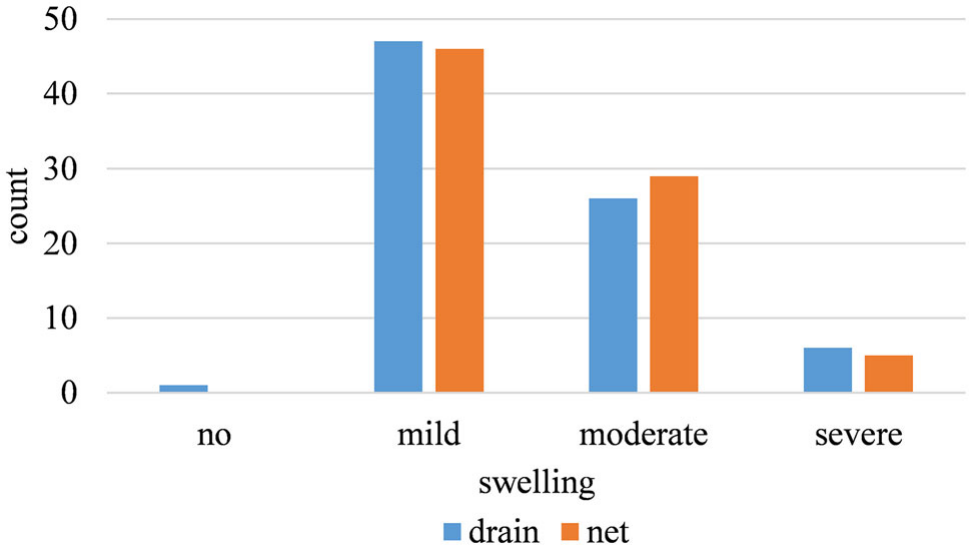
The incidence of swelling and its severity between both groups

No significant difference between both methods was found regarding the degree of postoperative edema. Most patients had mild edema (58.1%), while only 6.9% had a degree of edema described as severe or grade 3 (Fig. 2).

The degree of postoperative edema was found higher in revision surgery, where 60% of the cases who underwent revision facelifts were associated with moderate swelling, and 40% experienced marked edema. This was compared to the standard surgery where most patients experienced mild swelling (70.9%), (*P* = 0.000).

#### Secondary Outcomes

Other findings such as ecchymosis, congestion, necrosis, and other complications were also observed with no significant difference between both groups (Table 1).

**Table 1:** Descriptive analysis of the incidence of complications in relation to each method

	Drain	Net	Total	P value
Hematoma	0	1	1	0.295
	0.0%	1.3%	0.6%	
Seroma	7	7	14	
	8.8%	8.8%	8.8%	
Congestion	2	2	4	
	2.5%	2.5%	2.5%	
Ischemia	0 0	0 0	0 0	
	0.0%	0.0%	0.0%	
Pus	2	0	2	
	2.5%	0.0%	1.3%	
No complication	66	70	136	
	82.5%	87.5%	85.0%	

#### Ecchymosis

5% of all patients had significant postoperative ecchymosis, which was not statistically associated with either method (*P* = 0.468).

#### Congestion

Four cases developed congestion, with an equal proportion of two in each group, which refers to excessive fluid accumulation, especially blood within the subcutaneous tissue [23].

#### Necrosis

No incidence of ischemia or necrosis was reported among the study participants.

#### Skin Irregularities

There were few occurrences of neck irregularities, with no significant difference between the two groups. We believe this variation can be more related to poor skin undermining and irregular dissection under the skin flaps.

#### Infection

Two cases of pus collection were observed in the drain group with no incidence in the net group.

#### Correlation to Type of Surgery

**Fig. 3:**
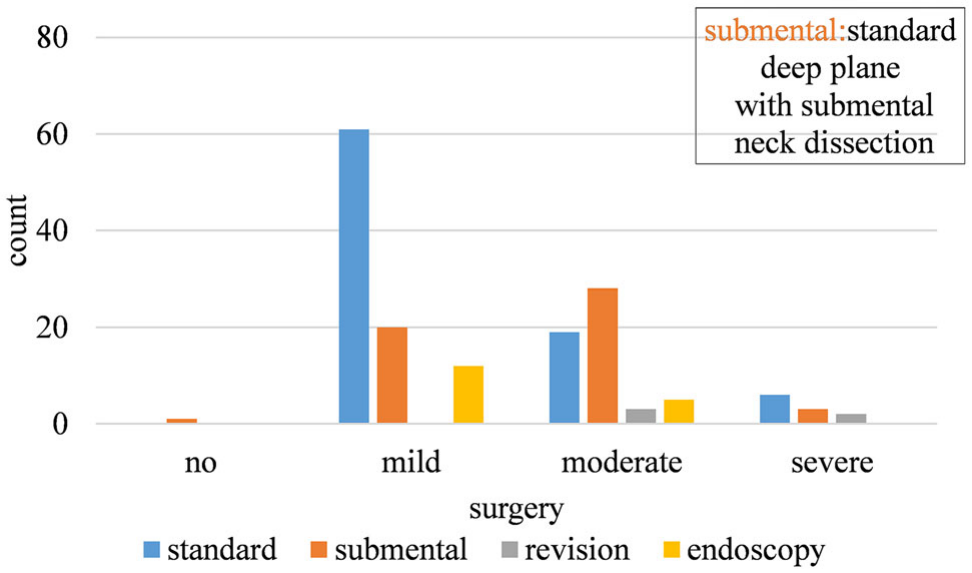
The relation between complications and type of surgery

Overall, the standard technique was associated with fewer complications: 7.0% in contrast to revision surgery which was associated with a 20.0% rate. In addition, patients who underwent neck dissection had a 30.8% complication rate (Fig. 3).

## Discussion

With the growing recognition of the deep plane facelift technique and the more courageous dissection under the platysma, creating more dead space and wider disturbance of the skin flap vascularity, the risk of developing complications has increased.

Hematoma has been the most feared complication after facelift surgery with the risk of returning to the operative theater and the stress added to the patient and the surgeon. However, it should be noted that the incidence of hematoma has varied in recent studies. It has been greatly reduced with the accurate identification of risk factors and the precise application of perioperative measures. In recent studies that included a large number of patients, this risk has been lowered to range from 0 to 9% [1–3] which reached 14% in older literature [9, 11]. Moreover, with the increased use of the sub-SMAS (the superficial musculoaponeurotic system) dissection technique, the tamponade action of the inelastic SMAS layer can limit the expansion of hematoma if developed under the SMAS. Therefore, further evidence is needed to assess the current risk with the use of recent modalities.

It should be noted that acute postoperative hematomas are usually large, and they require immediate evacuation in the operative theater by opening the sutures and cauterization of the bleeding vessels. They differ from delayed minor hematomas which are localized and can be up to 5 cm in diameter [9]. They are treated by aspiration or frequent drainage. It is hypothesized that by occluding the dead space, the hemostatic net can reduce the incidence of these tiny hematomas.

However, more precise evidence is needed to validate this assumption.

In addition to hematoma, the development of seroma and postoperative swelling are also significant concerns following facelift surgery. Seroma, which involves the buildup of serous fluid in the surgical site, can lead to delayed healing, infection, or discomfort if not addressed promptly [24, 25]. Similarly, we believe that postoperative edema has a negative impact on the recovery process, patient satisfaction, and surgical outcomes [19–21]. These complications highlight the importance of meticulous surgical technique and efficient postoperative care in reducing these risks.

Among the several methods used to decrease postoperative complications after facelift surgery, perioperative bleeding control is the most effective method. The literature showed that strict blood pressure control can decrease hematoma rates to 3.7% [26]. However, many studies have focused on assessing other adjunct methods for minimizing postoperative complications after facelift surgery. Thus, studies on the effectiveness of suction drains, hemostatic nets, compression dressings, and tissue sealants were brought into the literature. Most of these studies were retrospective. In addition, a significant contribution was made using meta-analysis; however, no research has strictly compared surgical drains with the newly introduced hemostatic net.

The majority of evidence focusing on drain use with facelift surgery does not conclude its superiority to other methods in minimizing postoperative complications. A study by Perkins et al. found that the placement of drains for 24 h postoperatively reduced seroma rates in the intervention group (15% versus 37%, *P* < 0.01), although the difference in hematoma rate between the two groups was not significant [6]. Jones and Grover in their study of 678 facelifts reached a similar conclusion, in which the incidence of hematoma did not improve with drain insertion in 229 patients in comparison with 449 patients without postoperative drains (4.8% versus 4.2%, *P* > 0.4). The use of drains, however, significantly decreased postoperative ecchymosis in the same research [27]. Jones et al. published the first prospective, randomized, controlled study assessing the effect of surgical drains after facelift surgery on 50 patients using a split face design, in which they found no statistically significant difference in hematoma or edema between drained and undrained sides (*p* = 0.56; *P* = 0.66) [16]. In addition, the benefit of drain use must be weighed against the downsides of being uncomfortable to the patient, and the fact that their removal may provoke bleeding.

The hemostatic net was introduced to facelift surgery by Andre Auersvald and Luiz Auersvald who were able to identify zero incidence of hematoma within the application of the net in comparison with a rate of 14.2 % detected in their control group [9].

Following the development of the net technique, Neto et al. retrospectively compared the incidence of hematoma while using the net in 100 consecutive surgeries to an average of 7.8% rate in two control groups. No cases of hematoma developed within the net group [12].

Furthermore, a retrospective cohort study of 663 facelifts revealed a significantly lower hematoma rate of 0.6% in the intervention group of patients for whom the hemostatic net was placed, compared to 3.9% in the control group (*P* = .006722) [11].

In addition, several studies concluded that the use of the hemostatic net decreased the hematoma rate significantly without increasing the possibility of ischemia or necrosis [9, 13, 14].

However, some studies demonstrate that the application of the net extends the surgery time by about 30 min; this highlights the importance of considering the added time and stress to the surgeon in future research. It is also important to note that the concomitant use of suction drains and the hemostatic net is not a rare practice. Although this technique can combine the safety of the net with the practical advantage of the drains allowing for fluid drainage and monitoring, the possibility of trapping the drain within the net sutures, causing pressure on skin flaps should be considered.

This study carefully examines the degree of postoperative swelling after the use of both methods, along with reviewing the incidence of other important postoperative risks, including hematoma and seroma rates.

The results demonstrate that no significant difference was found between both methods regarding the risk of hematoma and seroma formation. In addition, the severity of swelling among both groups was comparable. A significant association with complication rates was found in revision and neck lift surgeries.

We believe that the perioperative blood pressure control measures we adopted, combined with meticulous hemostasis, contributed to the low hematoma rate observed among our study participants. Our approach for that included maintaining an optimum systolic blood pressure of 120 mmHg before anesthesia, in addition to raising the blood pressure to the same optimum measure before undertaking a meticulous hemostasis. However, the relatively high seroma rate may be attributed to the commonly observed heavy necks in our Middle Eastern population which we addressed by neck dissection in 31% of the study population.

Although the evidence indicates that the hemostatic net can be a reliable method of decreasing postoperative complications after facelift surgery, more robust studies comparing patient-reported outcomes (PROs) to the significant patient discomfort caused by suction drains are needed. We suggest taking into consideration the possible added time and cost when applying the hemostatic net in future studies.

## Conclusion

The hemostatic net and surgical drain have comparable outcomes regarding  the risk of hematoma and seroma formation and the degree of postoperative swelling following deep plane facelift.

### Study Limitations

Including male participants in the study could have provided a more comprehensive analysis of complications risks and the effectiveness of each method in minimizing hematoma in such a high-risk group.

## References

[1] Baker DC, Stefani WA, Chiu ES. Reducing the incidence of hematoma requiring surgical evacuation following male rhytidectomy: a 30-year review of 985 cases. Plast Reconstr Surg. 2005;116(7):1973–87. 10.1097/01.prs.0000191182.70617.e9. 16327611 10.1097/01.prs.0000191182.70617.e9

[2] Ramanadham SR, Mapula S, Costa C, Narasimhan K, Coleman JE, Rohrich RJ. Evolution of hypertension management in face lifting in 1089 patients: optimizing safety and outcomes. Plast Reconstr Surg. 2015;135(4):1037–43. 10.1097/PRS.0000000000001131. 25811571 10.1097/PRS.0000000000001131

[3] Trussler AP, Hatef DA, Rohrich RJ. Management of hypertension in the facelift patient: results of a national consensus survey. Aesthet Surg J. 2011;31(5):493–500. 10.1177/1090820X11411292. 21719861 10.1177/1090820X11411292

[4] Grover R, Jones BM, Waterhouse N. The prevention of hematoma following rhytidectomy: a review of 1078 consecutive facelifts. Br J Plast Surg. 2001;54(6):481–6. 10.1054/bjps.2001.3623. 11513508 10.1054/bjps.2001.3623

[5] Maricevich MA, Adair MJ, Maricevich RL, Kashyap R, Jacobson SR. Facelift complications related to median and peak blood pressure evaluation. Aesthetic Plast Surg. 2014;38(4):641–7. 10.1007/s00266-014-0353-z. 24912427 10.1007/s00266-014-0353-z

[6] Perkins SW, Williams JD, Macdonald K, Robinson EB. Prevention of seromas and hematomas after face-lift surgery with the use of postoperative vacuum drains. Arch Otolaryngol Head Neck Surg. 1997;123(7):743–5. 9236595 10.1001/archotol.1997.01900070087014

[7] Tiourin E, Barton N, Janis JE. Methods for minimizing bleeding in facelift surgery: an evidence-based review. Plast Reconstr Surg Glob Open. 2021;9(8): e3765. 10.1097/GOX.0000000000003765. 34395151 10.1097/GOX.0000000000003765 PMC8360447

[8] Kamer FM, Nguyen DB. Experience with fibrin glue in rhytidectomy. Plast Reconstr Surg. 2007;120(4):1045–51. 10.1097/01.prs.0000278092.28351.c9. 17805134 10.1097/01.prs.0000278092.28351.c9

[9] Auersvald A, Auersvald LA. Hemostatic net in rhytidoplasty: an efficient and safe method for preventing hematoma in 405 consecutive patients. Aesthetic Plast Surg. 2013;38(1):1–9. 10.1007/s00266-013-0202-5. 23949130 10.1007/s00266-013-0202-5

[10] Da Rosa Rezende AR, Rezende KL, Chedid GB, Martins JMP, Collares MVM. A comparison of the efficacy of autologous fibrin glue/platelet-poor plasma versus suction drainage in preventing hematoma and seroma in rhytidectomy: a randomized, double-blind, controlled study. JPRAS Open. 2021;74(9):2290–5. 10.1016/j.bjps.2020.12.098. 10.1016/j.bjps.2020.12.098 33583759

[11] Janssen TJ, Maheshwari K, Sivadasan A, Waterhouse N. Hemostatic net in facelift surgery: a 5-year single-surgeon experience. Aesthet Surg J. 2023;43(10):1106–11. 10.1093/asj/sjad097. 37040449 10.1093/asj/sjad097

[12] Neto JC, Fernandez DER, Boles M. Reducing the incidence of hematomas in cervicofacial rhytidectomy: new external quilting sutures and other ancillary procedures. Aesthetic Plast Surg. 2013;37(5):1034–9. 10.1007/s00266-013-0084-6. 23408038 10.1007/s00266-013-0084-6

[13] Henry G, Auersvald A, Auersvald LA, Ospital C, Boucher F, Mojallal A. Skin perfusion after hemostatic net: an anatomic and radiologic study in a cadaver model. Aesthetic Surg J. 2024. 10.1093/asj/sjad286. 10.1093/asj/sjad286 37675581

[14] Kachare MD, Moore AC, Little J, O’Daniel TG. Response to: discussion of details in the application of laser-assisted fluorescence angiography with SPY-Q software analysis. Aesthetic Surg J. 2023. 10.1093/asj/sjad084. 10.1093/asj/sjad084 36996438

[15] Nahai F, Bassiri-Tehrani B, Santosa KB. Hematomas and the facelift surgeon: it’s time for us to break up for good. Aesthet Surg J. 2023;43(10):1207–9. 10.1093/asj/sjad225. 37437181 10.1093/asj/sjad225 PMC10501745

[16] Jones BM, Grover R, Hamilton S. The efficacy of surgical drainage in cervicofacial rhytidectomy: a prospective, randomized, controlled trial. Plast Reconstr Surg. 2007;120(1):263–70. 10.1097/01.prs.0000264395.38684.5a. 17572574 10.1097/01.prs.0000264395.38684.5a

[17] Jones BM, Grover R. Avoiding hematoma in cervicofacial rhytidectomy: a personal 8-year quest. Reviewing 910 patients. Plast Reconstr Surg. 2003;113(1):381–7. 10.1097/01.PRS.0000097291.15196.78. 10.1097/01.PRS.0000097291.15196.78 14707663

[18] Khansa I, Khansa L, Meyerson J, Janis JE. Optimal use of surgical drains: evidence-based strategies. Plast Reconstr Surg. 2018;141(6):1542–9. 10.1097/PRS.0000000000004413. 29608530 10.1097/PRS.0000000000004413

[19] Hochman B, Ferreira LM. Perioperative corticosteroids for preventing complications following facial plastic surgery. Cochrane Database Syst Rev. 2014. 10.1002/14651858.CD009697.pub2. 24887069 10.1002/14651858.CD009697.pub2 PMC11069365

[20] Weber M, Rahn J, Hackl M, et al. Postoperative swelling after elbow surgery: influence of a negative pressure application in comparison to manual lymphatic drainage: a randomized controlled trial. Arch Orthop Trauma Surg. 2023. 10.1007/s00402-023-04954-3. 37421514 10.1007/s00402-023-04954-3 PMC10491702

[21] D’Andrea F, D’Andrea L, Manzi E. Venoplant effect in the management of postoperative edema in plastic surgery: results of a randomized and controlled clinical trial. Aesthetic Plast Surg. 2018. 10.1007/s00266-018-1108-z. 29508020 10.1007/s00266-018-1108-z PMC5945761

[22] Marchac D, Greensmith AL. Early postoperative efficacy of fibrin glue in facelifts: a prospective randomized trial. Plast Reconstr Surg. 2005. 10.1097/01.prs.0000153219.32665.d5. 15731694 10.1097/01.prs.0000153219.32665.d5

[23] Merriam-Webster medical dictionary, 6th edition Springfield, MA; Merriam-Webster; 1995. https://www.merriam-webster.com/medical/. Accessed December 18, 2024.

[24] Kuroi K, Shimozuma K, Taguchi T, et al. Effect of mechanical closure of dead space on seroma formation after breast surgery. Breast Cancer. 2006;13(3):260–5. 10.2325/jbcs.13.260. 16929119 10.2325/jbcs.13.260

[25] Papanikolaou A, Minger E, Pais M-A, Constantinescu M, Olariu R, Grobbelaar A, Lese I. Management of postoperative seroma: recommendations based on a 12-year retrospective study. J Clin Med. 2022;11(17):5062. 10.3390/jcm11175062. 36078992 10.3390/jcm11175062 PMC9457167

[26] Bassiri-Tehrani B, Abi-Rafeh J, Baker NF, Kerendi AN, Nahai F. Systolic blood pressure less than 120 mmHg is a safe and effective method to minimize bleeding after facelift surgery: a review of 502 consecutive cases. Aesthet Surg J. 2023;43:1420–8. 10.1093/asj/sjad228. 37439229 10.1093/asj/sjad228

[27] Jones BM, Grover R. Avoiding hematoma in cervicofacial rhytidectomy: a personal 8-year quest reviewing 910 patients. Plast Reconstr Surg. 2004;113:381–7. 10.1097/01.prs.0000097291.15196.78. 14707663 10.1097/01.PRS.0000097291.15196.78

